# Localization of Dielectric Anomalies with Multi-Monostatic S_11_ Using 2D MUSIC Algorithm with Spatial Smoothing

**DOI:** 10.3390/s22145293

**Published:** 2022-07-15

**Authors:** Ahmad Bilal, Choon Sik Cho

**Affiliations:** Department of Electronic & Information Engineering, Korea Aerospace University, Goyang 10540, Korea; ahmadbilal@kau.kr

**Keywords:** antipodal Vivaldi antenna, Inverse Synthetic Aperture Radar (ISAR) imaging, near-field imaging, spatial smoothing

## Abstract

This article demonstrates that the complex value of S_11_ of an antenna, acquired in a multi-monostatic configuration, can be used for localization of a dielectric anomaly hidden inside a dielectric background medium when the antenna is placed close (~5 mm) to the geometry. It uses an Inverse Synthetic Aperture Radar (ISAR) imaging framework where data is acquired at multiple frequencies and look-angles. Initially, near-field scattering data are used for simulation to validate this methodology since the basic derivation of the Multiple Signal Classification (MUSIC) algorithm is based on the plain wave assumption. Later on, from an applications perspective, data acquisition is performed using an antipodal Vivaldi antenna that has eight constant-width slots on each arm. This antenna operates in a frequency range of 1 to 8.5 GHz and its S_11_ is fed to the 2D MUSIC algorithm with spatial smoothing whereas the antenna artifact and background effect are removed by subtracting the average S_11_ at each antenna location. Measurements reveal that this methodology gives accurate results with both homogeneous and inhomogeneous backgrounds because the size of data sub-arrays trades between the image noise and resolution, hence reducing the effect of inhomogeneity in the background. In addition to near-field ISAR imaging, this study can be used in the ongoing research on breast tumors and brain stroke detection, among others.

## 1. Introduction

Microwave imaging systems span a wide range of applications such as concealed weapon detection [1], medical imaging [2], through-the-wall detection [3], non-destructive testing [4], etc. In recent years, these systems have drawn significant attention owing to their lower cost and compact size. Although massive research is being carried out in these areas, commercially available solutions are still scarce mainly because of the unmet promises regarding robustness, size, and computational requirements [5].

Narrowing down to medical imaging, various multi-monostatic and multistatic systems have been proposed in the literature which use both Frequency Domain (FD) and Time Domain (TD) data. Mustafa et al. [6] have designed a multi-monostatic system for brain stroke detection where FD data is collected and converted to TD before pre-processing which is then fed to the Delay and Sum (DAS) algorithm. Mustafa [7] has analytically modeled a multi-monostatic system where TD backscattered data is computed using the Finite-Difference Time-Domain (FDTD) method and is processed by wavelet-matched filters. It is shown that the variance of the scalogram and Shannon wavelet entropy carries critical information about brain stroke detection. Mehranpour et al. [8] have introduced a novel hybrid background removal method to eliminate skin reflection and coupling between antennas. The authors have used Independent Component Analysis (ICA) for artifact response estimation and have employed a Wiener filter to suppress it. This enables their system to accurately localize a tumor that is just 5 mm in diameter. Ghimire et al. [9] have designed a novel feeding mechanism for a Vivaldi antenna array which is based on binary T-junction power splitters with un-even arm lengths. This design has a high gain and high directivity even at lower frequency ranges which is critical in localization systems. They use this array to transmit and receive UWB pulses for imaging a substrate material hidden inside a hollow spaced concrete block. A bistatic TD system has been designed by Shao et al. [10] that uses modulated Gaussian pulses. The received signal is fed to the Delay Multiply and Sum (DMAS) algorithm for image construction. In [11], a flexible electromagnetic cap has been designed that is embedded with 16 antenna elements and a matching layer. This is again a TD system that uses a 16-port Vector Network Analyzer (VNA). An interesting approach has been taken by Fedeli et al. [12] who have proposed a hybrid method for brain stroke detection. Their method uses synthetic aperture focusing, which is a qualitative method, along with the Inexact-Newton/Landweber method which is a quantitative method to accurately estimate the complex permittivity distribution inside the head phantom.

At this point, it is quite safe to say that most of the literature is inclined toward TD methods mainly because of the following reasons:

(1) Microwave imaging systems are analogous to radar systems where the transmitted signal is usually in the form of TD pulses [13,14].
(2) Constructed images are in TD so the TD evaluation of scattering mechanisms can be combined with the imaging algorithms [15,16].
(3) TD systems tend to offer faster scan times and lower costs of measurement devices [5].

Despite these advantages, some studies have been published that employ FD techniques. For instance, a multistatic system is proposed by Shao et al. [17] that uses the phase of $S_{21}$ in FD for image construction. The proposed method, referred to as “Phase Confocal Method (PCM)”, is similar to the DAS algorithm in a way that, instead of time shifts, it computes the phase shifts because of wave propagation. In 2019, Shao et al. [18] published another study that presents the Phase Shift and Sum (PSAS) algorithm and shows that the new method outperforms the DMAS algorithm. This calls for further investigation into the FD beamformers and the fact that both PCM and PSAS are multistatic FD methods leads us to the next logical step and which is to formulate a multi-monostatic FD method which is the subject of this article.

We employ the 2D MUSIC algorithm because it has been used with Radar Cross-Section (RCS) for ISAR imaging. However, our implementation is inspired by SpotFi [19] which uses wireless Channel State Information (CSI) in FD as the input data for the application of indoor localization of human targets. With respect to frequency, both RCS and CSI are different in the sense that RCS is a far-field quantity that is quasi-periodic while CSI follows different probability distributions depending on the environment, but the 2D MUSIC algorithm gives useful insights about the target using both data types. In addition to these quantities, in the field of antennas, *S*_11_ is a common parameter that represents the reflected power at the antenna port and is also known as the return loss. This quantity has been used by A. Y. Owda et al. [20] where it is demonstrated that a Synthetic Aperture Radar (SAR) framework can be employed for burn wound diagnostics. They perform a C-scan of the target geometry which is a common practice in Ground Penetration Radars (GPR) [21]. The acquired *S*_11_, for a frequency range of 15–40 GHz, is processed using Inverse Fast Fourier Transform (IFFT) for the detection of skin burns. Unfortunately, the distance between the sample and the antenna is not mentioned in the paper so it cannot be said whether it is a near-field or a far-field case. Furthermore, Trakic et al. [22] have proposed a Polar Sensitivity Encoding (PSE) scheme that uses antenna S-parameters collected in a multistatic configuration for the application of brain stroke detection. This warrants further investigation into the applicability of *S*_11_ with more advanced processing algorithms. In our implementation, we use a 2D MUSIC algorithm with data sub-array formation and feed it with $S_{11}$ of an antenna which makes it a multi-monostatic system that works with a single port VNA, hence, significantly reducing the amount of hardware by eliminating multiple antennas (usually ≥ 16) and RF switching circuitry. Furthermore, the heterogeneous background is considered, and spatial smoothing is applied to $S_{11}$ to minimize the image noise.

The rest of the paper is organized as follows. Section 2 presents the mathematical model for 2D MUSIC with special emphasis on spatial smoothing. Simulation results using both near-field scattering data and antenna *S*_11_ are given in Section 3 while Section 4 presents the measured results with both homogeneous and inhomogeneous background mediums. Section 5 concludes this article.

## 2. Mathematical Model

**Figure 1:**
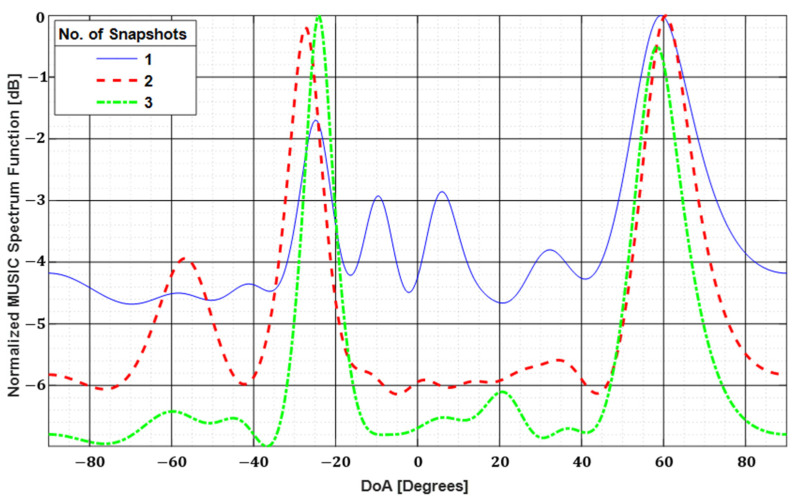
The 1D DoA estimation of two signals incident at −25° and 60°. Dynamic range and estimation accuracy are increased while side lobes are suppressed by using multiple snapshots of the incident signal.

In radar imaging, only a single snapshot of the signal is used for processing for real-time application [23]. This poses a serious problem in source localization as illustrated in Figure 1 where the MUSIC algorithm is employed for 1D Direction of Arrival (DoA) estimation of two signals incident at −25° and 60°.

The blue solid curve in Figure 1 shows the result of a single snapshot. It is quite clear that the side lobes at −10° and 6° will cause significant image noise which may shadow the original signal source in worst cases. The side lobe level decreases while the dynamic range is increased as we use a higher number of snapshots. However, to alleviate this problem, we use spatial smoothing which is a process of producing “virtual snapshots” by splitting the input field matrix into sub-matrices but it requires some workaround which is discussed in the context of near-field ISAR imaging.

### 2.1. Near-Field ISAR Imaging

**Figure 2:**
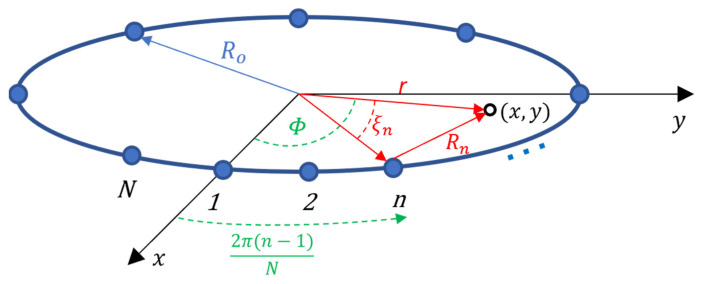
Geometry of an N-element uniform circular array with radius $R_{o}$. The point scatterer (x,y) is at a distance r from the phase center of the geometry.

Consider the scattered field for *m*th wavenumber $k_{m}$ and *n*th angle $\Phi_{n}$ which is given by

(1) $$E\left(k_{m},\Phi_{n}\right)=\int \int g\left(x,y\right)\;e^{2jk_{m}R_{n}\left(x,y,\Phi_{n}\right)}dxdy+u\left(m,n\right)$$

where g(x,y) is the reflectivity of the scatterer at (x,y) and $R_{n}$ is the distance between the nth antenna and the scatterer location (Figure 2) while u(m,n) is the additive white Gaussian noise with zero mean and variance $\sigma^{2}$.

For a uniform circular array of radius $R_{o}$, $R_{n}$ is given by the law of cosine,

(2) $$R_{n}\left(x,y\right)=\sqrt{r^{2}+R_{o}^{2}-2R_{o}r\cos\;\left(\xi_{n}\right)}$$

such that $\xi_{n}=\Phi -\frac{2\pi \left(n-1\right)}{N}$ and $r=\sqrt{x^{2}+y^{2}}$. For our application, we approximate $R_{n}$ as [24]

(3) $$R_{n}\left(x,y\right)\approx x\cos\;\Phi -y\sin\;\Phi -R_{o}+\left(x^{2}+y^{2}\right)/2R_{o}$$

and substitute in Equation (1) to obtain

(4) $$E\left(k_{x},k_{y}\right)=\int \int g\left(x,y\right)\;e^{2j\left(xk_{x}-yk_{y}\right)}\cdot e^{-2jkR_{o}}\cdot e^{2jk\left(x^{2}+y^{2}\right)/2R_{o}}dxdy+u\left(m,n\right)$$

where the scattered field has been transformed from (k,Φ) to ($k_{x},k_{y}$) grid and $k_{x}=k\cos\;\Phi$ and $k_{y}=k\sin\;\Phi$. Using vector notation, the discrete form of Equation (4) can be written as

(5) E=Ag+u

where,

(6) $$E={\left[E_{11}\;E_{21}\cdots E_{M1}\;E_{12}\;\cdots \;E_{MN}\right]}^{T}$$

(7) $$A=\left[a\left(x_{1},y_{1}\right)a\left(x_{2},y_{2}\right)\cdots a\left(x_{d},y_{d}\right)\right]$$

(8) $$g={\left[g_{1}g_{2}\cdots g_{d}\right]}^{T}$$

(9) $$u={\left[u_{11}\;u_{21}\cdots u_{M1}\;u_{12}\;\cdots \;u_{MN}\right]}^{T}$$

for a total of *M* frequencies, *N* antennas, and *d* scattering centers. Vector E is the total input data that is used by this method. a(x,y) are the steering vectors and are given by the exponential form of Equation (3).

(10) $$a\left(x,y\right)={\left[e^{2jkR_{1}},\ldots ,e^{2jkR_{n}},\;\ldots ,\;e^{2jkR_{N}}\right]}^{T}$$

To apply the 2D MUSIC algorithm in the near region, we obtain the correlation matrix $C_{xx}=\epsilon \left[EE{\;}^{*}\right]$ where the operator ϵ[·] represents the ensemble average, E is the measured field, and * denotes the complex conjugate transpose. Eigen-decomposition of $C_{xx}$ yields the signal and noise subspace where eigenvectors corresponding to (MN−d) smaller eigenvalues correspond to the noise subspace ***Z*** whose dimensions are MN×(MN−d). Using ***Z*** along with a(x,y), Equation (11) is used for a peak search. This is the MUSIC spectrum function in 2D which exploits the orthogonality of the signal and the noise subspaces.

(11) $$P_{MUSIC}\left(x,y\right)=\left|\frac{1}{a{\left(x,y\right)}^{*}\;ZZ^{*}\;a\left(x,y\right)}\right|$$

**Figure 3:**
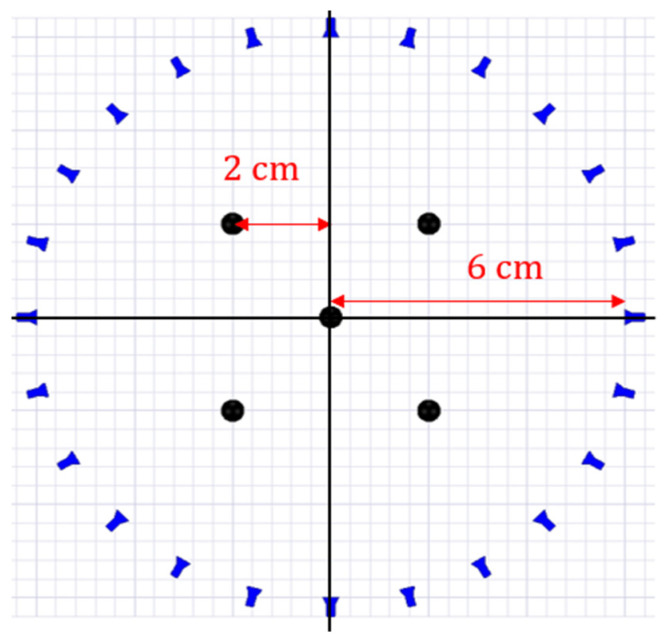
Simulated geometry of five point scatterers (black) and 24 antenna locations (blue). Each source emits a signal of 1–8.5 GHz and records a monostatic scattered field at its location.

So far, the basic near-field ISAR imaging approach is discussed which is a general case of far-field ISAR imaging because a closer look at Equation (4) shows that the first exponential term corresponds to the far-field region while the last two exponentials which contain the array radius $R_{o}$ correspond to the near region. Let us analyze the effect of these terms using an analytical simulation where the geometry of five point scatterers is illuminated by a source of 1–8.5 GHz placed at a distance of $R_{o}=6\;\mathrm{cm}$ from the phase center of the geometry which is at the origin. The geometry is rotated around its axis with a step size of 15° to emulate a synthetic aperture and monostatic scattered fields in the near region are collected at each location. Figure 3 shows the simulation setup.

**Figure 4:**
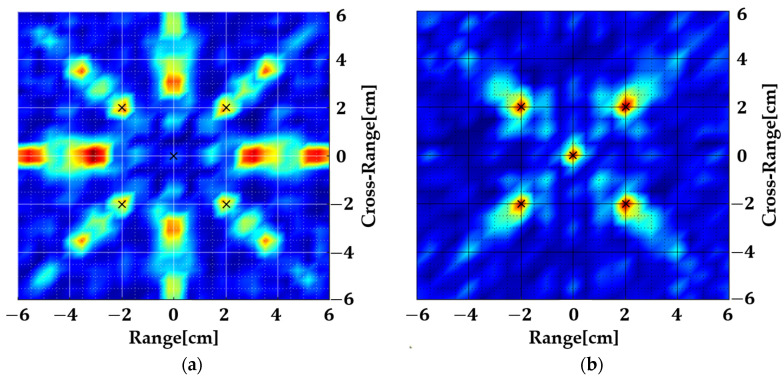

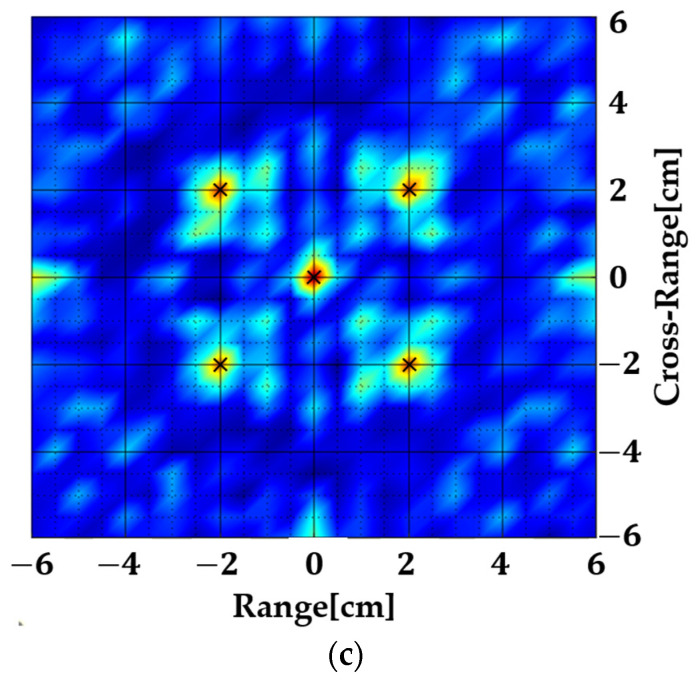
Near-field ISAR images of the geometry shown in Figure 3, (**a**) considering only the first exponential term, (**b**) considering the first two exponential terms, and (**c**) considering all terms.

The collected scattered field is fed to the 2D MUSIC algorithm where initially, only the far-field term is considered. The resulting image is shown in Figure 4a. Later on, the second term is also incorporated into the steering vector and the resulting image is shown in Figure 4b. Finally, all terms are included and the result is shown in Figure 4c.

The image shown in Figure 4a is as expected because the scattered fields are collected in the near region and only the far-field exponential is used without compensating for the spherical wavefront. Figure 4b shows that the targets are accurately localized even when only the second exponential is taken into account. Figure 4c is still accurate, but it shows that the contribution of the last term is minute. This is because the approximation of Equation (3) is a Taylor series expansion, and the last term has a negligible effect on the overall result. We ignore this term since it will produce non-separable terms in (x,y) and $\left(k_{x},k_{y}\right)$ which is critical for spatial smoothing.

Since $k=\sqrt{k_{x}{}^{2}+k_{y}{}^{2}}$ is still not separable in terms of $k_{x}$ and $k_{y}$ in the second exponential, i.e., $e^{-2jkR_{o}}$ but notice that it does not depend on the integration variables of Equation (4), hence, we take this term out and define

(12) $$\hat{E}\left(k_{x},k_{y}\right)=E\left(k_{x},k_{y}\right)\cdot e^{2jkR_{o}}$$

which can be carried out as a pre-processing step before eigen-decomposition.

**Figure 5:**
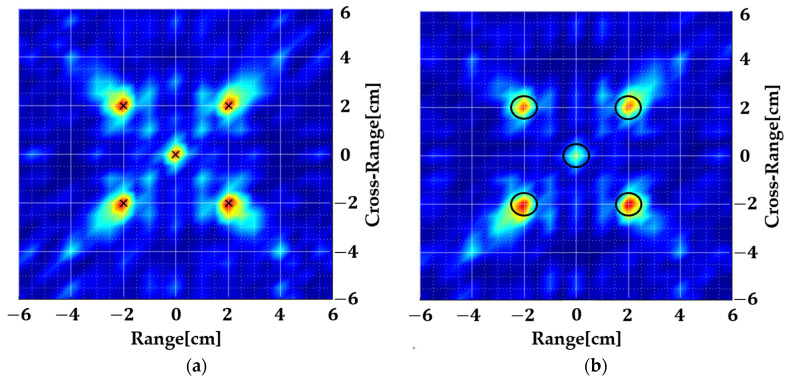
Near-field ISAR images of the geometry shown in Figure 3 by pre-processing using Equation (12) and using only the first exponential term. (**a**) $R_{o}=6\;\mathrm{cm}$ (**b**) $R_{o}=7\;\mathrm{cm}$ with target diameter changed to 1 cm. The method works with changing target size and array radius.

Figure 5 shows the result when instead of incorporating $e^{-2jkR_{o}}$ in the steering vector (Figure 4b), the scattered field is pre-processed using Equation (12) and then fed to the 2D MUSIC algorithm. There is a great deal of similarity between Figure 4b and Figure 5a which was expected but this step was mandated by the non-availability of separable terms in the steering vector. For further validation, using the same approach and simulation parameters, another case is run where the scattered near-field is computed using a full-wave simulation. The target size is increased to 1 cm, and the array radius is 7 cm. The resulting image is shown in Figure 5b which still accurately localizes all targets. Hence, it can be established that this method can be used for the detection of targets in the near region of a uniform circular array.

The approach discussed so far uses the scattered near-field data (shown in Equation (6)) all at once. This is efficient in terms of computation time, but the images contain significant noise which can be critical when the targets are inside an inhomogeneous background. We discuss in the next section how we can split the data into sub-arrays to increase the SNR.

### 2.2. Near-Field ISAR Imaging with Spatial Smoothing

To benefit from spatial smoothing, the data and the steering vectors are arranged in a specific order which is discussed here. Initially, the scattered field matrix shown in Equation (6) is arranged as

(13) $$E=\left[\begin{array}{ccc} E_{11}\;\;\;E_{12}\;\;\;E_{13} & \cdots & E_{1N}\\ \vdots & \ddots & \vdots \\ E_{M1}\;\;\;E_{M2}\;\;\;E_{M3} & \cdots & E_{MN} \end{array}\right]$$

Next, Equation (12) is used to compensate for the effect of the spherical wavefront and $\hat{E}$ is computed which is of the same size as the matrix E. Based on the trade-off between resolution and background noise, we split matrix $\hat{E}$ into (M−m+1)×(N−n+1) sub-matrices each of size m×n and rearrange them as shown in Equation (14).

(14) $${\hat{E}}_{1}=\left(\begin{array}{c} {\hat{E}}_{11}\\ {\hat{E}}_{12}\\ \vdots \\ {\hat{E}}_{1n}\\ \vdots \\ {\hat{E}}_{m1}\\ {\hat{E}}_{m2}\\ \vdots \\ {\hat{E}}_{mn} \end{array}\right);{\hat{E}}_{2}=\left(\begin{array}{c} {\hat{E}}_{12}\\ {\hat{E}}_{13}\\ \vdots \\ {\hat{E}}_{1(n+1)}\\ \vdots \\ {\hat{E}}_{m2}\\ {\hat{E}}_{m3}\\ \vdots \\ {\hat{E}}_{m(n+1)} \end{array}\right)\ldots {\hat{E}}_{T}=\left(\begin{array}{c} {\hat{E}}_{\left(M-m+1)(N-n+1\right)}\\ {\hat{E}}_{\left(M-m+1)(N-n+2\right)}\\ \vdots \\ {\hat{E}}_{(M-m+1)N}\\ \vdots \\ {\hat{E}}_{M(N-n+1)}\\ {\hat{E}}_{M(N-n+2)}\\ \vdots \\ {\hat{E}}_{MN} \end{array}\right)$$

where T=(M−m+1)×(N−n+1) is the total number of sub-arrays. It should be noted that the size of each sub-array is smaller than the total matrix $\hat{E}$ since the number of frequencies and look-angles are limited to a smaller value (i.e., *m* < *M* and *n* < *N*). Since these data have less frequency and angular bandwidth than the total data, the resulting images lose some resolution, but on the bright side, allow us to form a total of *T* auto-correlation matrices. We compute the new auto-correlation matrices of all ${\hat{E}}_{T}$ sub-arrays and take their mean to average out the effect of false scatterers or inhomogeneity which may shadow the actual anomaly in the constructed image.

(15) $$C_{xx}=\frac{1}{T}\overset{T}{\underset{i=1}{\sum }}{\hat{E}}_{i}{\hat{E}}_{i}^{*}$$

**Figure 6:**
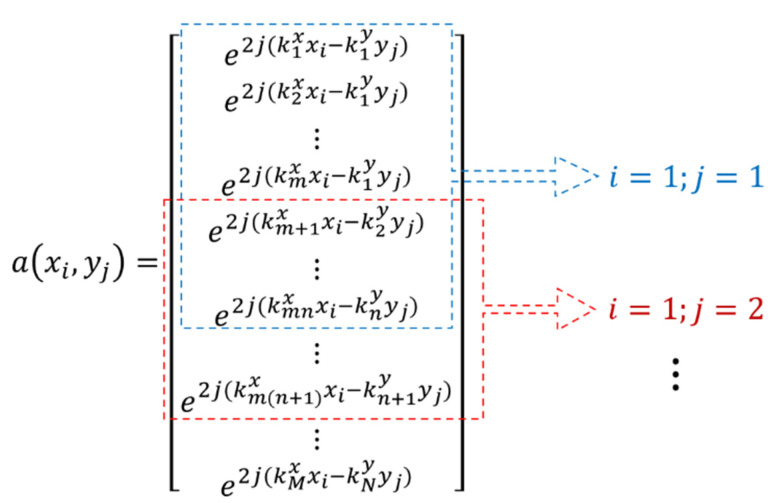
Steering vectors of length mn are taken out of the total vector length MN for computation of each pixel.

The new correlation matrix $C_{xx}\;$ has a size of (mn×mn) which would have been (MN×MN) without the smoothing process. Eigen-decomposition of $C_{xx}$ yields the signal and noise subspaces where the eigenvectors corresponding to (mn−d) smaller eigenvalues correspond to the noise subspace Z whose dimensions are (mn×(mn−d)). The variable d is a parameter of the MUSIC algorithm that defines the number of signals [25]. Since the size of the noise subspace has been reduced because of spatial smoothing, the size of the steering vector a(x,y) has to be adjusted accordingly and swept across the imaging domain so that the 2D MUSIC spectrum function (Equation (11)) could be computed. Figure 6 shows the arrangement of the steering vectors for computation of each image pixel while Algorithm 1 summarizes the proposed method in the form of a pseudocode.

In Figure 6, *k* is the wavenumber while the superscripts *x* and *y* show the *x* and *y* component, respectively. Subscript *m* and *n* are the sizes of the sub-array chosen for spatial smoothing while *M* and *N* are the total number of frequencies and aspect angles as defined previously. For every pixel $\left(x_{i},y_{j}\right)$, the corresponding steering vector is computed and inserted in Equation (11) to calculate the spectrum function. Although this methodology can be used for general near-field ISAR imaging, we use it to localize a dielectric anomaly inside a dielectric background medium.
**Algorithm 1: 2D MUSIC with Spatial Smoothing**
**Input:** Complex matrix of size *M* × *N* as shown in Equation (13)
**Output:** Near-field ISAR image with target location**1**Compute ***k_x_*** and ***k_y_*** using frequencies and angles;**2**Translate ***E***(*k_m_*, *Φ_n_*) to ***E***(*k_x_*, *k_y_*) using polar to cartesian transformation;**3**Compute ***Ê***(*k_x_*, *k_y_*) using Equation (12);**4**Rearrange into sub-arrays using Equation (14);**5**Compute *C_xx_* using Equation (15) and construct matrix ***Z*** whose columns are the eigenvectors of *C_xx_* corresponding to eigenvalues that are smaller than the threshold *d*;**6**
**for***each sub-array***7**

**for***each pixel***8**


Initialize steering vector ***a***(*x*, *y*);**9**


Fill ***a***(*x*, *y*) with corresponding entries according to Figure 6;**10**


Compute image pixel using ***Z*** and ***a***(*x*, *y*) in Equation (11);**11**

**end****12**
**end****13****Plot***P_MUSIC_*(*x*, *y*)

## 3. Simulation

### 3.1. Using Scattered Field in Near-Region

**Figure 7:**
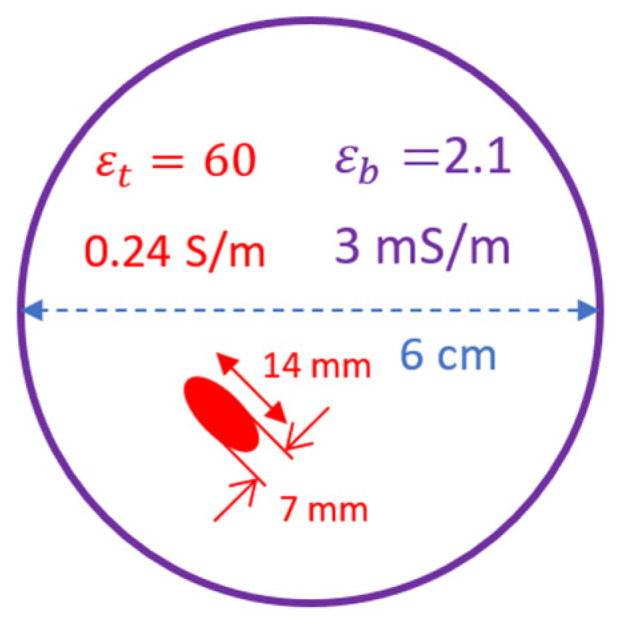
Geometric parameters and electrical properties of the simulated geometry.

Our implementation of this method employs near-field scattering with spatial smoothing for noise reduction. We feed it with near-field scattering data collected at a distance of 3 cm (reactive near-region) from the edge of the background medium. Figure 7 shows the simulated geometry where an elliptical target, shown in red, is immersed inside a cylindrical background (shown in purple). 

**Figure 8:**
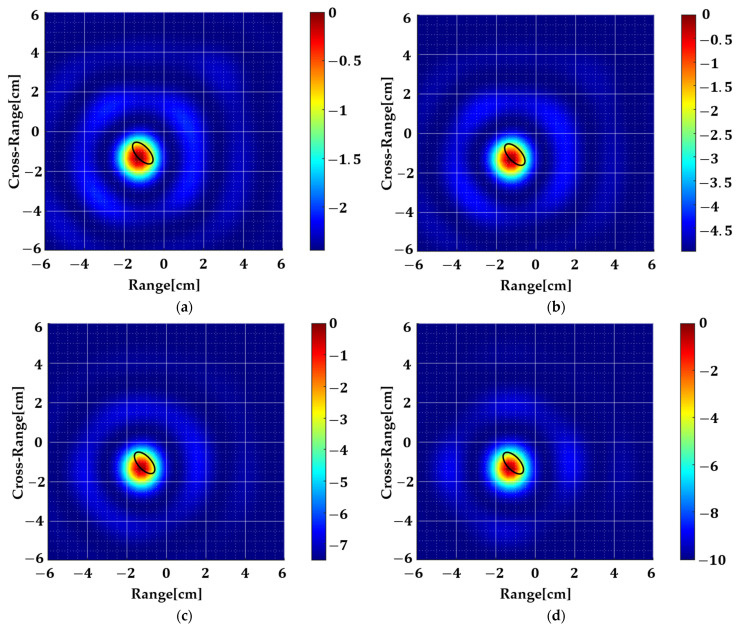
Simulated images of the geometry shown in Figure 7. The black ellipse is the true target location. (**a**) is the output of basic 2D MUSIC where *m* = *M* and *n* = *N*. (**b**) *m* = (*M* − 2) and *n* = (*N* − 2). (**c**) *m* = (*M* − 4) and *n* = (*N* − 4). (**d**) *m* = (*M* − 6) and *n* = (*N* − 6). By increasing the number of sub-arrays, the dynamic range increases as shown in the color bar.

The major and minor radii of the target are 7 mm and 3.5 mm, respectively, with a dielectric permittivity of 60 and conductivity of 0.24 S/m. The radius of the background medium is 3 cm while its permittivity and conductivity are 2.1 and 3 mS/m, respectively. Using point sources at a distance of 3 cm from the geometry, we illuminate it from all sides with a step size of 15° and for a frequency range of 1–4 GHz. The monostatic, near-field scattering data are computed, and the mean is subtracted to remove the background artifact. Then it is fed to the algorithm discussed in Section 2. Figure 8a shows the result obtained from the basic 2D MUSIC spectrum function. The important thing to note is that the dynamic range (DR) in this image is just 2.4 dB. This shows that the SNR is quite low but nevertheless, information about the true location is still present in the input data. We exploit this fact and use spatial smoothing to improve the SNR. By splitting the input data, as given by Equation (14), and computing the auto-correlation matrix by averaging individual sub-arrays, we reduce signal noise before eigen-decomposition. Sequentially increasing the number of sub-arrays, as shown in Figure 8b–d, increases the dynamic range (~10 dB), hence, making the system less error-prone.

Furthermore, it should be mentioned that although the SNR has been improved, there is an associated trade-off and that is the computation time which increases non-linearly with the number of sub-arrays. The simulations in Figure 8a–d are conducted on Intel i5-8500 CPU with a clock speed of 3 GHz and the computation time is 7.5, 43, 70, and 90 s, respectively.

**Figure 9:**
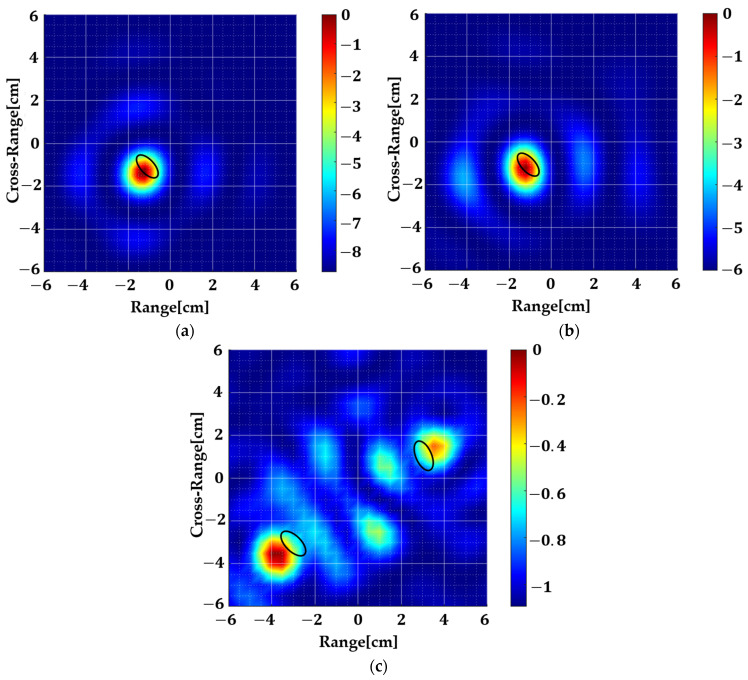
Analysis of the geometry shown in Figure 7 with varying contrast. The dielectric permittivity of the target is varied (**a**) $\varepsilon_{t}=10,\;\varepsilon_{b}=2.1,\;\sigma_{t}=0.24\;S/m,\;\sigma_{b}=3\;\mathrm{mS}/m$, (**b**) $\varepsilon_{t}=3,\;\varepsilon_{b}=2.1,\;\sigma_{t}=0.24\;S/m,\;\sigma_{b}=3\;\mathrm{mS}/m$, and (**c**) $\varepsilon_{t}=10,\;\varepsilon_{b}=2.1,\;\sigma_{t}=0.5\;S/m,\;\sigma_{b}=0.1\;S/m$. The dynamic range decreases with contrast but it can be tuned by varying sub-array sizes. Furthermore, this method has a limitation if there are multiple targets.

For analysis purposes, the permittivity of the target is changed to reduce the contrast and study its effect. It can be seen in Figure 9b that this method works well even when the target dielectric $\varepsilon_{t}=3$ which is very close to the background dielectric $\varepsilon_{b}=2.1$. Although there is some penalty in the form of reduced dynamic range since the reflection is not strong enough in this case, it can be tackled by changing the sub-array size. Furthermore, this method has a limitation when multiple targets are present (Figure 9c) which is possibly because of the multipath effect. This aspect is being left as future work.

There is also another aspect that should be highlighted from a measurement perspective. Although these results made it clear that FD data, acquired in the near region, holds valuable information about the hidden target, measuring near-field for practical use is still a challenging task which limits the application of this method. It is a well-known fact that any geometry, when placed in the near region of an antenna, affects its $S_{11}$, and since measuring $S_{11}$ is a much simpler task, we investigate if it holds the same information about the geometry. We use the complex value of $S_{11}$ because we are interested in the range-cross range profile of the geometry rather than the insertion loss of the antenna. Since $S_{11}$ of an antenna is typically below −10 dB, the importance of spatial smoothing greatly increases.

### 3.2. Using Scattering Parameter

**Figure 10:**
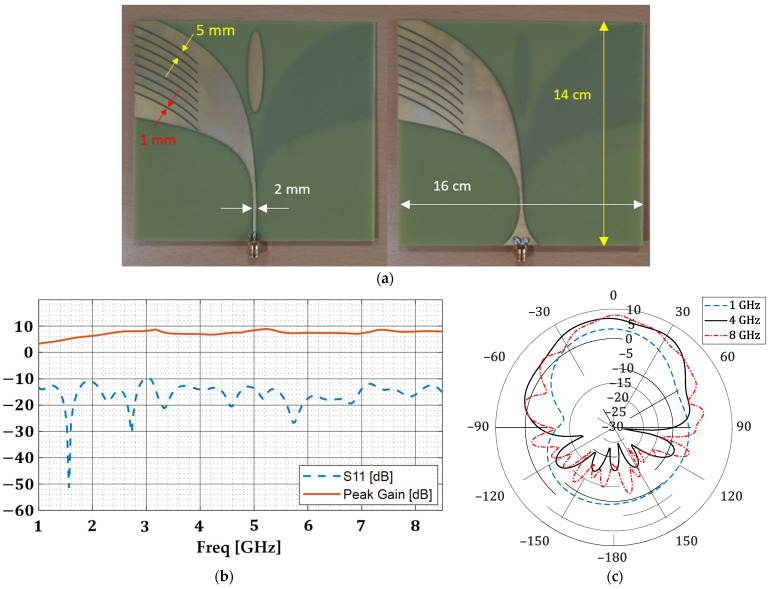
(**a**) Top and bottom layer of the fabricated antipodal Vivaldi antenna. (**b**) Antenna $S_{11}$ and peak gain in dB. (**c**) Antenna gain (dB) in the H-plane.

We conduct full-wave simulations where an antipodal Vivaldi antenna is designed to illuminate the target geometry. The antenna is a conventional edge-fed design on an FR-4 substrate that has 1 mm constant-width slots on both layers. This type of design is chosen because of its directivity and higher gain. Figure 10a shows the fabricated antenna while Figure 10b,c show the $S_{11}$, peak gain and radiation pattern in dB.

**Figure 11:**
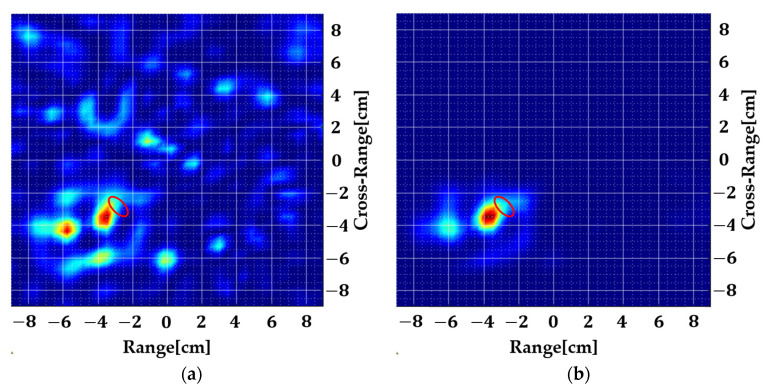
Simulated images of the dielectric anomaly where the red ellipse is the true location. (**a**) Without spatial smoothing and (**b**) with spatial smoothing (*m* = *n* = 5).

The antenna is placed at a distance of 5 mm from the geometry and its $S_{11}$ is recorded for an all-aspect scan with a step size of 15°. The antenna is operated at a frequency range of 1–8.5 GHz and only 63 points are taken. The size of the total data set is 63 × 24 which is very small for accurate localization of such a small anomaly. The algorithm is tested in both scenarios, i.e., with and without spatial smoothing. In the latter case, simulation time is significantly reduced because all the data are used at once but as evident from the results, strong clutter may appear which, in an experimental setting, may shadow the original target. Figure 11 shows the computed images.

The simulated $S_{11}$ is fed to the 2D MUSIC algorithm to obtain Figure 11. A significant amount of clutter appears even in a simulated environment but still, even without spatial smoothing, the immersed target is localized. Next, Equation (14) is used to split the data into sub-arrays where m=n=5 is chosen which gives a total of 36 sub-arrays. Averaging over all the sub-arrays eliminates the image noise which may prove to be a useful tool in reducing the effect of measurement imperfections and heterogeneous background which is evaluated in the next section.

## 4. Measured Results

**Figure 12:**
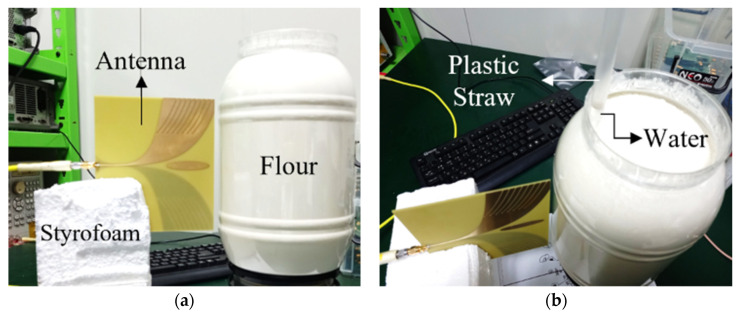
Measurement setup: (**a**) Side view where the antenna is mounted in a block of Styrofoam. (**b**) A plastic straw of 1 cm diameter filled with mineral water $(\varepsilon_{t}=80$) is inserted in the background medium which constitutes wheat flour ($\varepsilon_{b}=2.5$ ).

Several measurements were conducted to validate this methodology. The designed antenna, shown in Section 3, was used to illuminate a geometry comprising of a plastic container filled with wheat flour ($\varepsilon_{b}=2.5$) to emulate the background medium while a plastic straw was filled with mineral water ($\varepsilon_{t}=80$) and placed in that background to represent the dielectric anomaly. The diameter of the container, in the imaged plane, was 16.7 cm while the plastic straw had a diameter of 1 cm which is even smaller than the simulation. This emulates the tumor development in its very early stages. The antenna was connected to an Agilent E5071B network analyzer and was kept static while the geometry was rotated around its axis. Figure 12 shows the measurement setup where the antenna is mounted on a block of Styrofoam while the plastic container is placed at a distance of 5 mm from the antenna aperture. For all measurements, the antenna orientation is kept vertical so that a vertical polarization could be achieved. A frequency sweep of 1–8.5 GHz was used while an all-aspect scan was performed with a step size of 15°. The collected $S_{11}$ was processed offline using MATLAB.

### 4.1. Homogeneous Background

**Figure 13:**
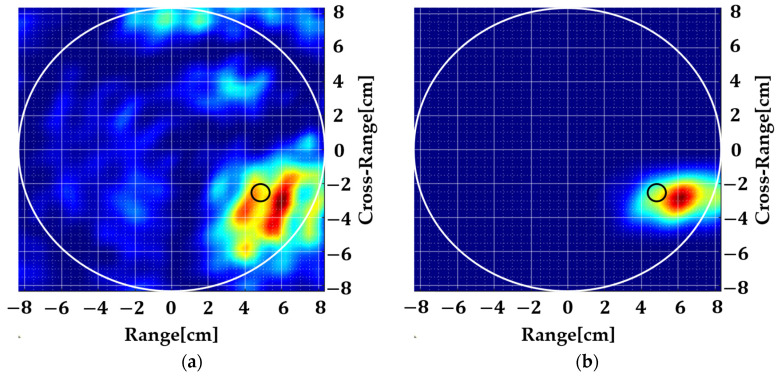

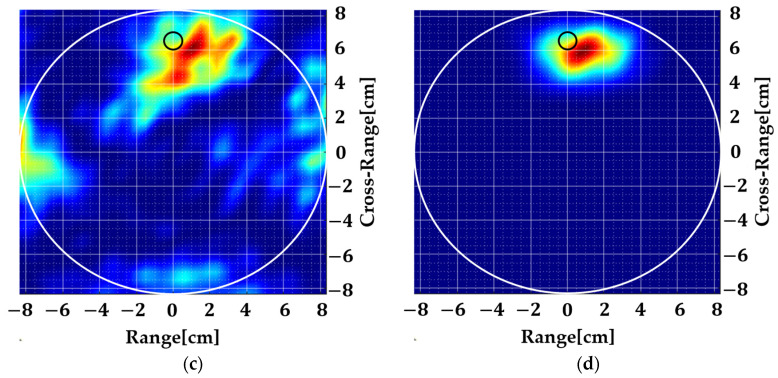
Measured results with a homogeneous background. The white circle shows the boundary of the background while the small black circle shows the size and location of the anomaly: (**a**) Experiment 1 without spatial smoothing, (**b**) with spatial smoothing, (**c**) Experiment 2 without spatial smoothing, (**d**) with spatial smoothing.

Several measurements were conducted where only flour was used in the background to emulate homogeneity. The resulting images of the two experiments are shown in Figure 13. It can be seen that the localization of the dielectric anomaly is quite accurate in all cases. Figure 13a,b shows the same measurement where the target is 2.5 cm deep inside the background. Some image noise is appearing in Figure 13a which is removed through spatial smoothing. In Figure 13c,d, the target is kept 1.35 cm deep inside the background. It is clear that even a small anomaly of 1 cm diameter distorts the antenna $S_{11}$ in a way that it can be localized using an FD method, unlike its TD counterpart, where target size is of great importance because of their direct dependence on a strong reflection for the purpose of ranging. A comparison with the existing literature is given in Table 1.

### 4.2. Inhomogeneous Background

**Figure 14:**
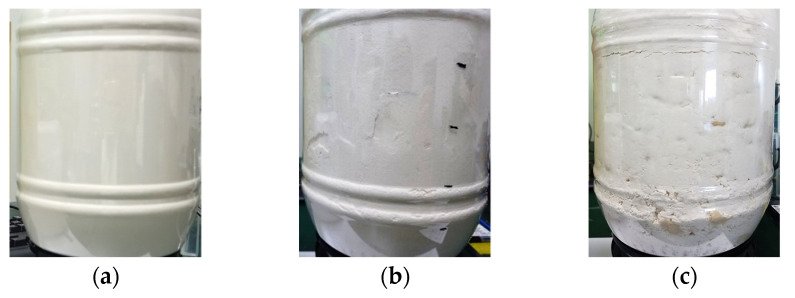

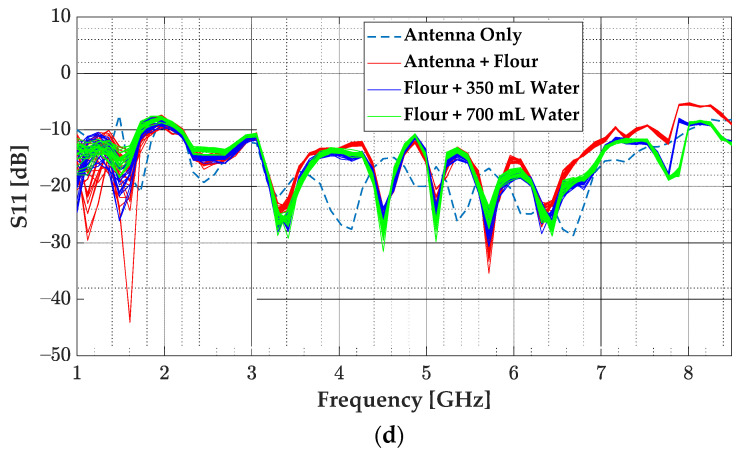
Inhomogeneous medium after adding water to the background: (**a**) flour only, (**b**) flour + 350 mL water (10%), (**c**) flour + 700 mL water (20%), and (**d**) antenna $S_{11}$ in three cases.

Using the same measurement setup, we conducted two more experiments where 350 mL and 700 mL of water were added to the background which amounts to 10% and 20% of the total volume, respectively. Figure 14a–c shows the container with and without the inhomogeneity while Figure 14d shows the measured $S_{11}$.

Adding 350 mL of water with flour formed a more granular mixture that does not sit in a very homogeneous fashion in the container as shown in Figure 14b. Further adding 350 mL of water gave it a dough-like texture while concentrating more water in certain locations as seen in Figure 14c. Rotating this structure in front of the antenna changed its $S_{11}$, as shown in Figure 14d, where higher frequencies are more affected by the inhomogeneity.

**Figure 15:**
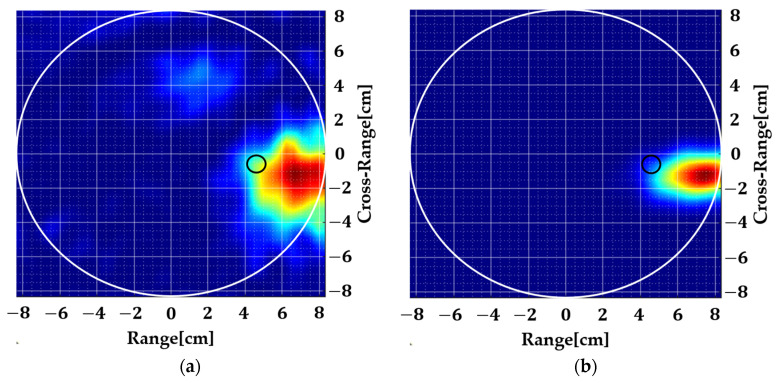

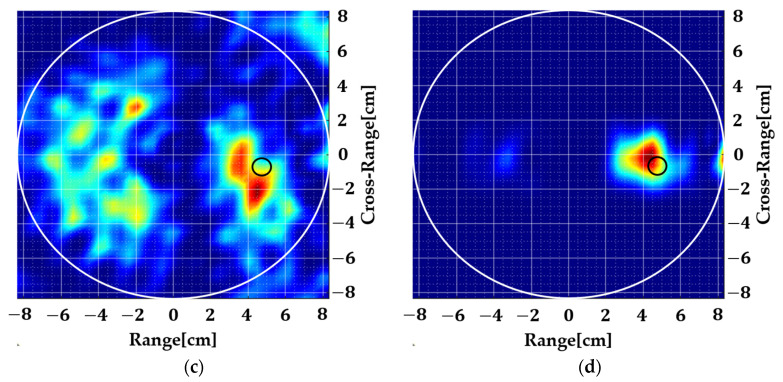
Measured results with an inhomogeneous background. The small black circle shows the true target location where background contains: (**a**,**b**) 10% inhomogeneity, (**c**,**d**) 20% inhomogeneity.

Figure 15 shows the resulting images where it is evident that more amount of inhomogeneity in the background yields more image noise as expected (Figure 15c). Applying spatial smoothing to the input data removed this noise to a great extent. Considering a realistic scenario of the human brain, by volume, less than 10% of blood is present in the background. Our results show that this can be achieved using a simple multi-monostatic prototype using a 2D MUSIC algorithm.

## 5. Conclusions

An ISAR imaging-based framework is applied in this paper that uses a 2D MUSIC algorithm with spatial smoothing to localize very small dielectric anomalies hidden inside a dielectric background. It is shown that the raw $S_{11}$ of an antenna can be directly fed to the algorithm. Full-wave simulations are conducted to verify this approach and it is shown that spatial smoothing can be used for image noise reduction while preserving the true target location. For further validation, a simplistic prototype is designed where an antipodal Vivaldi antenna is used for data collection which is processed offline. Results show that the proposed methodology works effectively in both homogenous and inhomogeneous backgrounds which can be very noisy at times. The strength of this method lies in the tunable nature of image noise which can be controlled by adjusting the sub-array size. This work shows that a single antenna/single port measurement system can deliver useful insights about a hidden dielectric anomaly even in a complex scenario where the background medium contains highly dispersive inhomogeneity. In future work, advanced background removal techniques can be studied as a pre-processing step for this method such that the targets hidden inside an asymmetric background could be localized. In addition, multipath mitigation methods could be incorporated to implement this method for the accurate localization of multiple targets. Furthermore, this methodology should be tested with more directive antennas, possibly arrays, so that the properties of radiating elements could be related to this method. This work can be used for near-field ISAR imaging with wide apertures for the early detection of brain hemorrhagic regions and breast tumors.

## Figures and Tables

**Table 1:** Comparison with available imaging methods and systems.

Reference	Antenna Configuration/Locations	Method	Bandwidth	Anomaly Diameter (mm)
[6]	Monostatic (16 and 32 antennas)	TD	1–4 GHz	28 × 14
[7] (Simulated)	Monostatic (14 antennas)	TD	1.5 and 2 GHz	7.2 × 6.3 × 3.6
[8]	Multistatic (37 antennas)	TD	2.2–13.5 GHz	5
[18]	Multistatic (2 antennas; 19 × 24 locations)	FD	2–7 GHz	19.5 and 17 × 10 × 12
[26]	Multistatic (160 Antennas)	Quantitative Imaging	0.9–1.8 GHz	39 × 23 × 23
This Work	Monostatic (24 locations)	FD	1–8.5 GHz	10

## Data Availability

Data available on reasonable request from the corresponding author.
